# Surface wax in the ancestral grapevine *Vitis sylvestris* correlate with partial resistance to Powdery Mildew

**DOI:** 10.1186/s12870-023-04311-x

**Published:** 2023-06-07

**Authors:** Xinshuang Ge, Birgit Hetzer, Christine Tisch, Andreas Kortekamp, Peter Nick

**Affiliations:** 1 Molecular Cell Biology, Botanical Institute, Karlsruhe Institute of Technology, Fritz-Haber-Weg, 76131 Karlsruhe, Karlsruhe, Germany; 2 Max Rubner-Institut (MRI) - Federal Research Institute of Nutrition and Food, Karlsruhe, Germany; 3 DLR Rheinpfalz State Education and Research Center of Viticulture and Horticulture and Rural Development, Neustadt an der Weinstraße, Germany

**Keywords:** Appressoria, Epicuticular wax, *Erysiphe necator*, Grapevine genetic resources, Powdery Mildew, *Vitis sylvestris*

## Abstract

**Background:**

Powdery Mildew of Grapevine belongs to the major diseases in viticulture and requires intensive use of fungicides. Genetic introgression of resistance factors from wild grapes from North America and, recently, China, has been successful, but wine made from those varieties is still confronted with low consumer acceptance, due to differences in taste.

**Results:**

The current work explores the potential of *Vitis vinifera sylvestris*, the wild ancestor of domesticated Grapevine, with respect to containing *Erysiphe necator*, the causative agent of Powdery Mildew. Making use of a germplasm collection comprising the entire genetic variability remaining in Germany, we show that there is considerable genetic variation in the formation of leaf surface waxes exceeding wax formation in commercial varieties.

**Conclusions:**

High wax formation correlates with reduced susceptibility to controlled infection with *E. necator* linked with perturbations of appressoria formation. We propose *V. vinifera sylvestris* as novel source for resistance breeding since it is genetically much closer to domesticated grapevine than the hitherto used sources from beyond the species barrier.

**Supplementary Information:**

The online version contains supplementary material available at 10.1186/s12870-023-04311-x.

## Highlights

The European Wild Grape (Vitis sylvestris) harbours genetic factors that stimulate surface waxes on the leaf and impede the infection by Erysiphe necator, the causative agent of Powdery Mildew.

**Supplementary Information:**

The online version contains supplementary material available at 10.1186/s12870-023-04311-x.

## Background

Grapevine (*Vitis vinifera*) is among the fruits with the highest cash value with an annual output of around 1,250 billion €(*Organisation International du Vigne et du Vin* [1]. This productivity comes with a certain price – *Vitis vinifera* belongs to the crops with the highest demand for plant protection, especially against fungal diseases. Just in the US, the fungicide consumption is in the range of around 90 million € per year [2]. For Germany, a recent estimate [3] arrives at 1400 € total costs, per hectar, which for the whole country sums up to around 140 millon € per year. This susceptibility to diseases derives fromhuman selection favouring fast growth and sweet fruits, which was compensated by a progressive loss of stilbenes, the major phytoalexins in grapevine [4]. Furthermore, *V. vinifera* was something like a glacial relict that had survived the Pleistocene in refugia, such as the Rhone delta in Southern France or the Caucasus region [5–7]. Therefore, *V. vinifera* is a naïve host for biotrophic fungi that had co-evolved with wild *Vitis* species in North America and succeeded in invading Europe during the second half of the nineteenth century (for a recent review see [8]). Both, Downy Mildew, caused by the oomycete *Plasmopara viticola*, and Powdery Mildew, caused by the ascomycete *Erysiphe necator*, sweeped in epidemic waves all over the viticultural areas of the planet (for review see [9]). Both diseases require numerous treatment cycles: in Europe, 7–8 cycles are common [10, 11], which in some years, such as 2021, can easily double. This intensity of chemical plant protection will not only cause higher labour costs and compacted soils, but also fuels the evolution of resistant pathogen strains (for review see [12]). Moreover, synthetic fungicides accumulating in the environment exert a very negative ecological footprint (for a review specific for viticulture see [13]).

The search for better alternatives must start with a thorough understanding of the lifecycle to identify potential Achilles’ Heels, where a specific strategy might attack. *Erysiphe necator* is a biotrophic pathogen [9, 14] infecting both sides of leaves, but also the surface of inflorescences and berries. Starting from small and diffuse white colonies, the infection can rapidly progress to entire leaves or bunches and also spread to the abaxial side of the leaves. The berries crack open in a characteristic manner and soon start to rot due to secondary infections by molds. Moreover, the taste of infected berries is not palatable, such that already a low degree of infection renders the entire yield unsuited for wine making. The infection starts from cleistothecia that are formed late during summer and attach to crevices in the bark. Alternatively, the fungus can enter developing buds, and the mycelium overwinters inside to emerge in the next spring when the bud breaks open [15]. After release, the conidia germinate on the surface of the leaves and form appressoria to penetrate through the cuticle into the epidermis scavenging the nutrients, needed for further development. Rapidly, the mycelium covers the entire surface. As soon as a few warm days follow in sequence, aerial hyphae protrude and form, by a rapid asexual cycle, new conidia that are spread by the wind and launch a new round of infections.

To contain Powdery Mildew, hygienic measures, such as improving ventilation by adjusting plant structure by appropriate pruning and defoliation of the berry zone in summer, help to a certain extent [16]. Likewise, biocontrol by mycophagous mites and hyperparasites has been demonstrated to be effective to some extent [17, 18]. However, the use of synthetic fungicides is still the dominating approach with all negative consequences, such as the evolution of resistant pathogen strains. Moreover, synthetic fungicides accumulate in the environment and impact ecology. The same holds true for copper used in organic viticulture (for a review, also on the economic aspects, see [19]). Since wild *Vitis* species from North America have co-evolved with *E. necator*, they harbour a strain-specific resistance that qualifies as true Effector-Triggered Immunity [20]. By introgression into *V. vinifera*, new varieties with resistance to Powdery Mildew have been bred that allow to reduce fungicide load by more than 80% [21]. The identification of new resistance loci in wild grapes from China is extending the genetic repertory. For instance, overexpression of WRKY factors from the Chinese wild species *V. pseudoreticulata* can confer resistance linked with activation of salicylic acid signalling [22] and accumulation of proanthocyanidins [23]. Since these Chinese grapes lack any co-evolutionary history with the fungus, the resistance of these new genetic resources is certainly of a different nature (for a critical discussion see Jiao et al*.*, 2016). This qualitative difference is very valuable for resistance breeding, though, since the resistance can be rendered more durable by gene stacking [21, 24, 25].

While the search for resistance factors against Powdery Mildew has been focussed on wild species from North America and China, the wild ancestor of Grapevine, the European Wild Grape (*V. vinifera ssp. sylvestris* in the following designated as *V. sylvestris*) has been neglected so far. The potential of this Crop Wild Ancestor is illustrated by studies, where some genotypes were found to be partially resistant to Downy Mildew [4], and to fungi causing Grapevine Trunk Diseases [26, 27]. Indeed, several genotypes of *V. sylvestris* show a partial resistance upon inoculation with *E. necator* [28], motivating a more systematic study of the underlying mechanisms that, like those of the Chinese wild grapes, are unlikely to be due to effector-triggered immunity.

The first step of infection is the formation of an appressorium requiring physical interaction with the host surface. Alterations of this host surface are expected to interfere with this interaction step leading to a delay of colonisation, which would give the host more time to deploy defence. For instance, leaf hairs can significantly impair infection of pathogens because they reduce the wettability of the surface [29, 30]. A further factor controlling the physical contact are surface waxes. In fact, their importance as barriers to ward off abiotic and biotic stresses is well established (for recent reviews see [31, 32]). The morphology of epicuticular waxes is variable – different microstructures including filaments, platelets, crystals, and tubules are known and often coexist [32]. These microstructures are not random by-products but often convey specific functions, illustrated by the famous Lotus Effect, where wax nanostructures on the leaf surface generate a self-cleaning surface [33]. The wax microstructures depend on the chemical composition that will enable specific routes of self-assembly but are also guided by structural cues from the leaf as shown by studies, where surface waxes have been removed and then allowed to regenerate, such that their formation could be followed by Atomic Force Microscopy [34]. The functional relevance of these structures, their dependence on chemical composition, and the presence of guiding cues strongly support the view that the microstructure of surface waxes is under genetic control.

While the spatial cues have remained elusive, the mechanisms controlling the chemical composition of surface waxes have been dissected quite intensively. Epicuticular waxes are lipophilic complex mixtures of compounds with very long carbon chains, mainly odd-numbered ketones and secondary alcohols or even-numbered primary alcohols, esters, and fatty acids [35]. The reason for the numbering is that all these components originally derive from Very Long Fatty Acids (VLCFAs) that are even-numbered because they are generated by condensation of acetyl-CoA building blocks. The synthesis of the ketones and the secondary alcohols involve a terminal de-carboxylation step by CYP96A15, resulting in a carbon chain that is odd-numbered [36]. Wax synthesis starts in the plastid, where fatty acids with chain lengths up to C_18_ are generated and then transported into the ER, where they are prolonged to the VLCFAs that can reach up to C_34_ (for review see [37]). How these very lipophilic molecules reach the plasma membrane, is matter of debate (for a review see [38]). Based on ultrastructural data, a physical interaction of ER and plasma membrane has been suggested. Alternatively, Lipid Transfer Proteins (LTPs) were proposed to move the long-chained components to the plasma membrane. However, based on cuticular phenotypes of *Arabidopsis* mutants impaired in vesicle traffic, Golgi and Trans-Golgi-Network seem to be required [39]. On the other hand, there is a membrane passage requiring an ABC transporter [40], which is not compatible with simple exocytosis. In the apoplast, two LTPs are needed to bridge the distance from the plasma membrane to the final destination at the outer surface of the apoplast [41, 42].

Since surface waxes are likely to affect appressorium formation, and since *V. sylvestris* seems to harbour genetic factors that confer partial resistance to *E. necator* [28], we wondered, whether more pronounced wax structures might be involved in *V. sylvestris*. We used a germplasm collection comprising the entire known gene pool still available in Germany for this ancestor of domesticated Grapevine [43], most of whose genomes had been fully sequenced [44], such that this collection could be subdivided into, phylogenetically defined, clades. We explored leaf surface waxes in this collection using Cryo Scanning Electron Microscopy (CryoSEM) followed by quantitative image analysis. We observed a large variability that differs between the different phylogenetic clades of German *V. sylvestris*, whereby most genotypes produce more pronounced wax layers as compared to commercial varieties of *V. vinifera.* Comparing the abundance of surface waxes with susceptibility to *E. necator* upon controlled infection, we find a significant increase, if the abundance of surface waxes drops below a threshold. Comparative time-course studies of early infection on hosts differing in the abundance of their surface waxes show that appressorium formation is perturbed and delayed on a host with pronounced wax structures as compared to the commercial variety which shows only a very scarce wax layer. We arrive at a model, where this delay in early development might give the host a time gain, in which it can deploy defence before it can be quelled by fungal effectors contributing to a partial resistance.

## Results

### Surface wax structures depend on genotype and on cell differentiation in *Vitis*

**Fig. 1:**
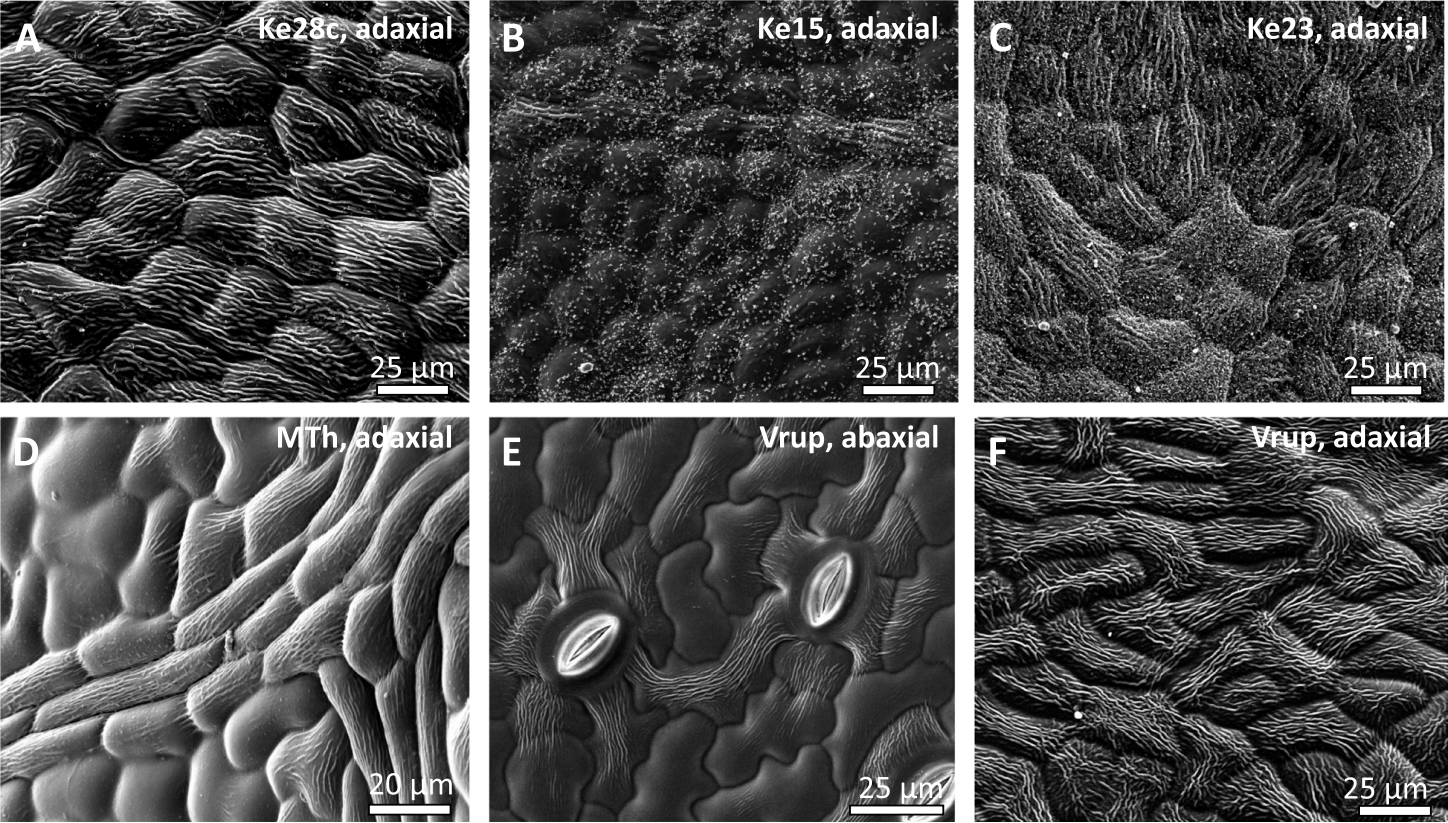
Surface-wax structures depend on genotype and on cell differentiation in *Vitis*. Representative images obtained by Scanning Electron Microscopy from fully expanded leaves. **A-C** Different structures of surface waxes from intercostal regions at the adaxial side to illustrate different structures of surface waxes, such as **A** long wax wings in *V. sylvestris* Ke28c, **B** small wax crystals in *V. sylvestris* Ke114, C mixed occurrence of long wax wings and small wax crystals in *V. sylvestris* Ke23. **D-F** Cell type differences of surface waxes. **D** Long wax wings are found along the epidermal cells covering leaf veins but are absent from intercostal regions in the adaxial side in *V. vinifera* Müller-Thurgau. **E, F** Surface waxes differ between the abaxial (**E**) and the adaxial (**F**) side in *V. rupestris*

To get a survey on the variability of surface-wax structures in *Vitis*, we investigated a set of 115 grapevine accessions by Cryo Scanning Electron Microscopy (CryoSEM). The set contained mainly accessions from *V. vinifera sylvestris*, the wild ancestor of domesticated Grapevine, along with a couple of commercial and traditional varieties (Suppl. Table 1). We examined both, adaxial and abaxial sides of fully expanded leaves and were able to discern three types of wax structures. Most prominent were long wing-like structures often aligning with the long axis of epidermal cells (Fig. 1A). In some cases, they were replaced by small wax crystals (Fig. 1B), and often both structures occurred together (Fig. 1C). We noted that cell differentiation had an influence on the incidence of these surface-wax structures. The long wing-like structures were frequent on the epidermal cells covering leaf veins but were absent from intercostal regions in the adaxial side (Fig. 1D). Generally, the surface waxes were less developed at the abaxial side, where they mainly lined the accessory cells of the stomatal complex (Fig. 1E). In contrast, the wax structures were most abundant in the pavement cells of the adaxial side (Fig. 1F). Since the development of wax structures obviously depended on genotype and on cell differentiation, we focused for the subsequent analysis on adaxial pavement cells.

### Surface wax forms depending on leaf development

**Fig. 2:**
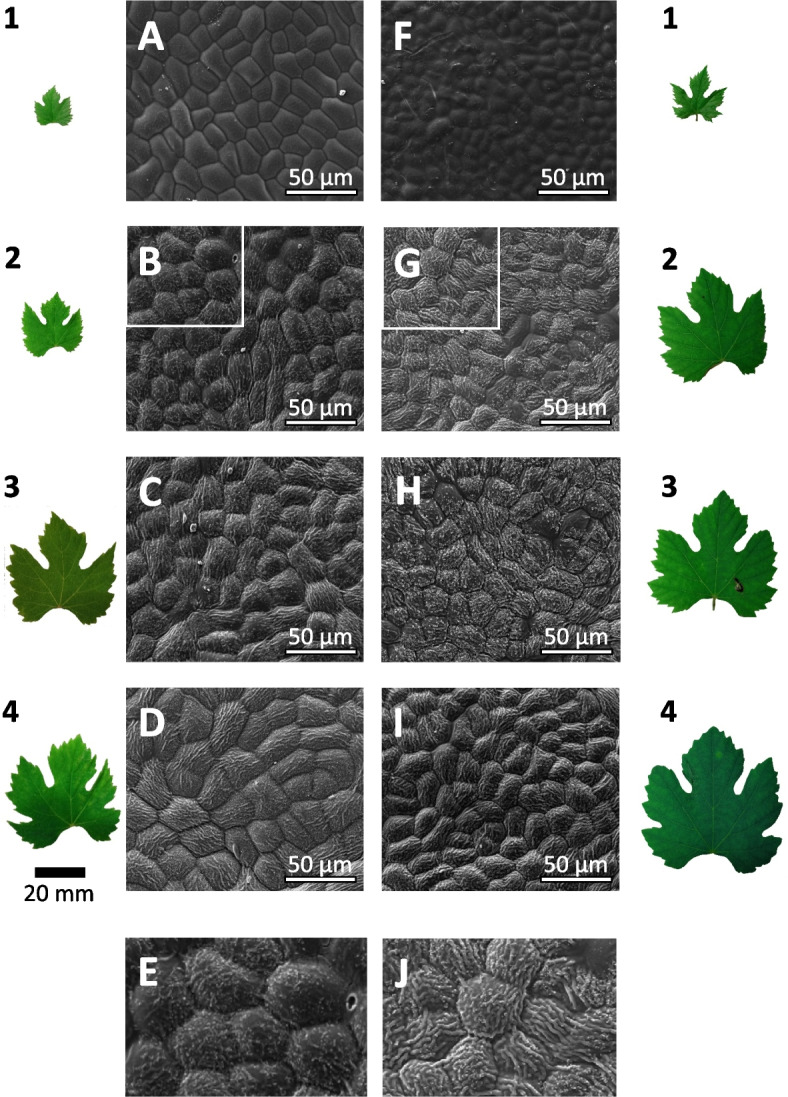
Surface-wax formation depends on leaf development. Representative images obtained by Scanning Electron Microscopy from the adaxial surface of leaves at different position from the apex in *V. sylvestris* Ke35 (**A-E**) versus Ke114 (**F-J**). Representative specimens of the leaves are shown in parallel to visualise the progress of leaf expansion. Zoom-ins from leaf #2 (white squares in **B, F**) are shown in **E** and **J** to show the different abundance of wax wings between the two genotypes

As to prepare the screen of the entire population, we first assessed the temporal progression of wax formation depending on leaf development. When we followed surface wax structures from the shoot axis to the base, we found them clearly more pronounced in the more developed leaves towards the base (Fig. 2). However, differences between genotypes became manifest already from the second leaf onwards, as exemplarily shown for *V. sylvestris* Ke35 (Figs. 2B, E) and Ke114 (Figs. 2G, J). The more abundant wax formation in very young leaves of *V. sylvestris* Ke114 (Figs. 2G, J), remained stable over the subsequent leaf stages (Figs. 2H, I). However, *V. sylvestris* Ke35, where surface waxes were not very prominent in the very young leaves (Figs. 2B, E), displayed increasing wax formation in the subsequent leaf stages (Figs. 2C, D), such that in the fourth leaves, the difference between the genotypes had almost vanished (compare Figs. 2D and 2I). Based on these preparatory studies, it was clear that any comparative study needs to define the developmental states of the leaves. Since leaf expansion differs considerably between the genotypes, it was not possible to use leaf area as criterion – a leaf of a given area from a sylvestris genotype might be already mature with respect to wax formation, while the same area in a leaf of ‘Müller-Thurgau’ might still be in full expansion. We, thus, decided, to use the first fully expanded leaves (leaf 4) for the comparative screen. Furthermore, since wax formation turned out not to be a qualitative trait, we decided to quantify wax abundance by quantitative image analysis.

### Time course and amplitude of surface wax formation shows genetic variation

**Fig. 3:**
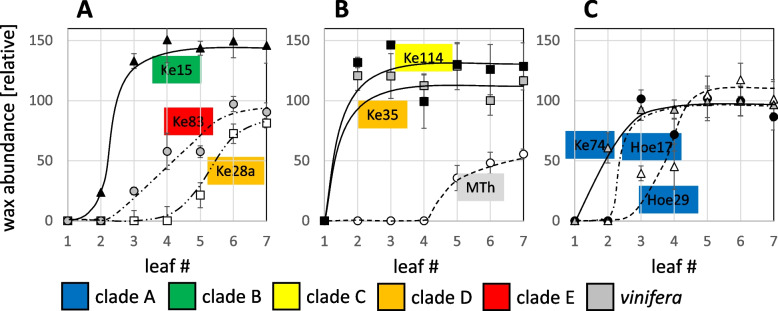
Time course of surface-wax formation in representative genotypes from different clades of the *sylvestris* population (as defined in Suppl. Fig. S2) along with the *vinifera* variety Müller-Thurgau. Adaxial wax was determined from SEM images based on quantitative image analysis in subsequent leaves starting from newly formed leaves (#1). Full expansion was achieved from #5. Data represent mean values and SE from 3 individual leaves per data point

To get insight into the role of genetic factors, we followed surface-wax formation over time in nine grapevine genotypes estimating wax abundance by quantitative image analysis (Suppl. Fig. S1). It should be kept in mind that this approach is only semi-quantitative yielding relative values that can be compared within an experimental series (such as developmental time courses of different genotypes collected in parallel), but cannot be used across experimental series, for instance, conducted in different years. We compared *vinifera* variety ‘Müller-Thurgau’, which is of economic relevance in South Germany to representatives of the European Wild Grapevine population, sampling individuals from all phylogenetic clades that had been inferred from a genome-wide comparison of Single-Nucleotide Polymorphisms (Suppl. Fig. S2). While all *sylvestris* genotypes accumulated wax earlier and to higher abundance as compared to ‘Müller Thurgau’ (Fig. 3), there was considerable variation, even between representatives from the same clade. For instance, Ke35 had already reached a maximal level from leaf 2 (Fig. 3B), while Ke28a, belonging to the same clade, did not produce any significant wax structures till leaf 4 and even remained significantly lower at leaf 7 (Fig. 3A). The high levels seen in Ke35 were even excelled by wax formation in Ke15 (Fig. 3A), and in Ke114 (Fig. 3B). In some cases, genotypes belonging to the same clade reached similar final levels of surface wax, but clearly differed with respect to the onset of wax accumulation. While in Ke74, already leaf two had almost attained its maximal level, Hö17 initiated this one leaf later (very swiftly, however), and in Hö29, two more leaves were required to see significant wax formation (Fig. 3C). Thus, the formation of surface wax is clearly linked with the genotype. Since the time courses of accumulation differed, we decided to use leaves that had just completed their expansion to compare wax levels across the population.

### Genetic variation for surface wax accumulation in *V. sylvestris*

**Fig. 4:**
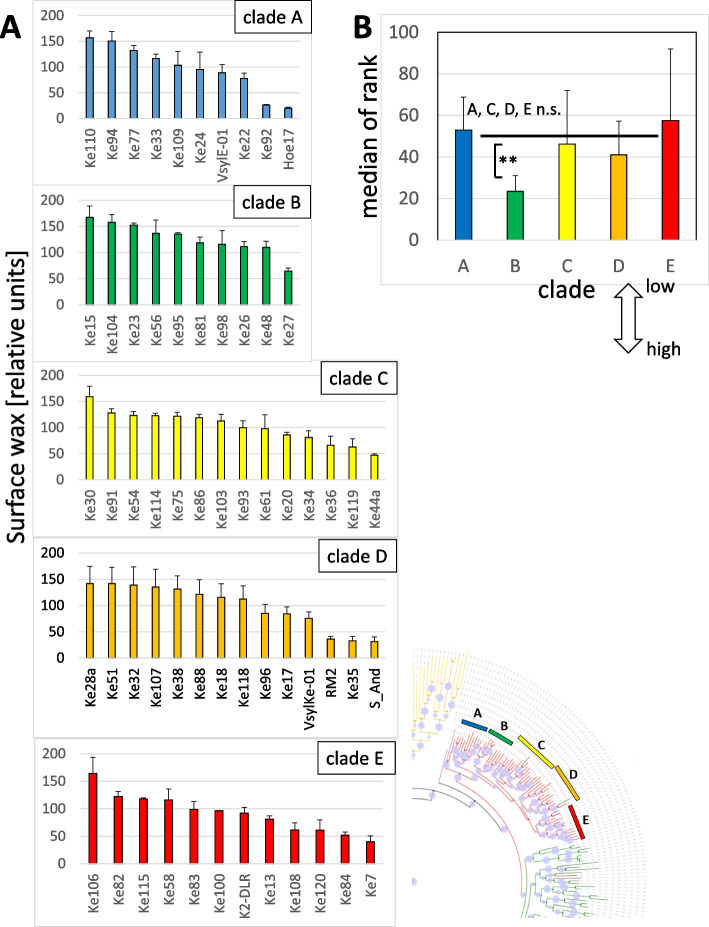
Genetic variability of surface-wax formation within the different clades of the *sylvestris* population (as defined in Suppl. Fig. S2). The different genotypes were cultivated side by side, summer 2017 in the Botanical Garden of the KIT. Data represent mean values and SE from 3 individual leaves per genotype collected from fully expanded leaves (#6). **A** Wax abundance for the individual genotypes of clades A-E. **B** clade differences in wax accumulation. All genotypes were pooled and ranked with respect to their wax accumulation. Data represent median and quartiles for each clade. Note that the median for clade B is significantly (*P* < 1%) lower as compared to the other clades based on a non-parametrical Kruskal–Wallis test meaning that clade B overall accumulates more wax than the other clades

To get insight into genetic variability of surface wax accumulation in *V. sylvestris*, the ancestor of domesticated grapevine, we probed fully expanded leafes in sixty genotypes originating from the Rhine peninsula Ketsch, representing the last viable population in Central Europe. For these sixty genotypes, molecular phylogeny based on the entire genomes was available [44]. This allowed to discern five, phylogenetically defined, clades A to E (Suppl. Fig. S2). The wax abundance within individual clades varied considerably (Fig. 4A). For instance, in clade A, genotype Ke110 accumulated more than 6 times more wax than Hö17. Similar differences were encountered also in the other clades. To test for significant differences, we pooled all 115 genotypes of this study (Suppl. Table 1) and ranked them according to the measured wax levels. This ranking approach was necessary, because the quantification of wax abundance by quantitative image analysis yields only relative values that can be compared within an experimental series, but are not transferrable between different measuring campaigns, conducted, for instance, in different years. Moreover, the values for wax abundance are skewed and do not follow a normal distribution. While wax accumulation of the clade A, C, D, E were statistically not different, clade B had a significantly lower median (based on a non-parametrical Kruskal–Wallis test, *P* < 0.01). This means, that wax accumulation was significantly higher in this clade as compared to the others (Fig. 4B).

### Genotypes with low wax content are more susceptible to Powdery Mildew

**Fig. 5:**
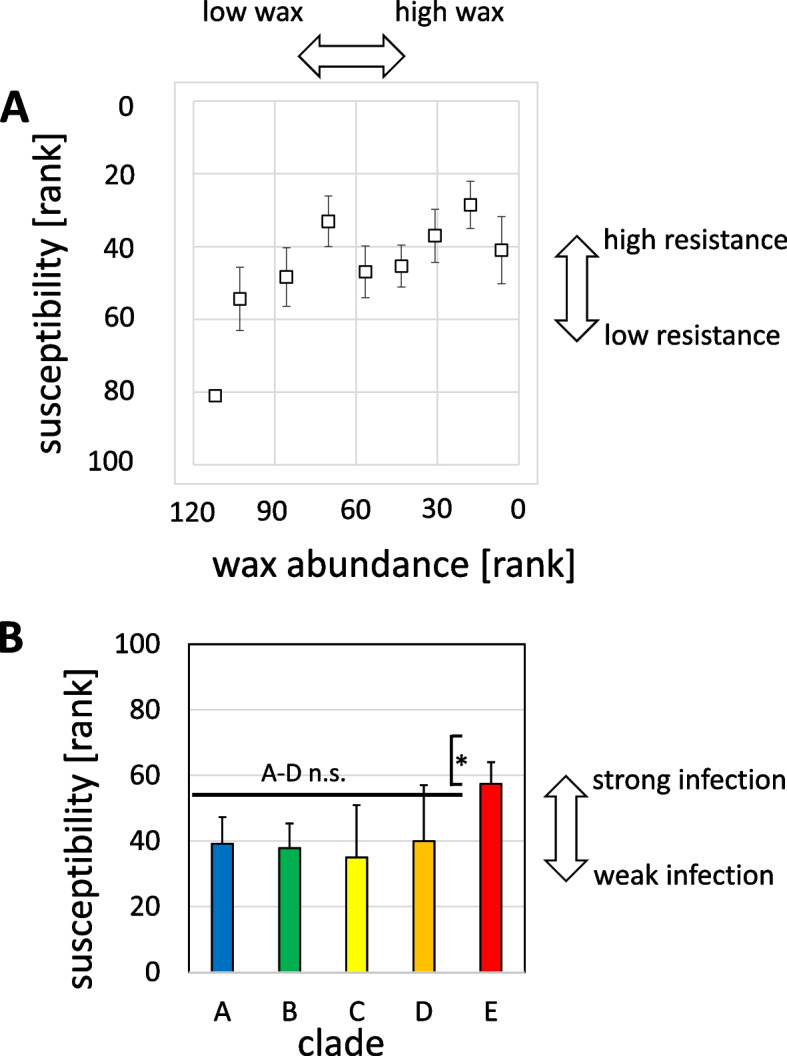
Relationship between susceptibility to Powdery Mildew and abundance of surface waxes in the *sylvestris* population. **A** Susceptibility over wax abundance using a ranking system (susceptibility was scored by a bonitation system, therefore, a non-parametrical approach was used to probe for correlations) For each genotype, the rank in susceptibility (increasing numbers mean increasing susceptibility) and wax abundance (increasing numbers mean decreasing wax abundance) were determined and plotted. Data represent median and quartile values from pools of ten genotypes following in the ranking list for wax abundance. **B** Susceptibility of the different clades of the *sylvestris* population (as defined in Suppl. Fig. S2). Data represent median and quartiles for susceptibility of all genotypes belonging to the respective clade. * significance at *P* < 0.05 based on a non-parametrical Kruskal-Wallice test

The causative agent of Powdery Mildew, *Erysiphe necator*, infects grapevine leaves by forming an appressorium that penetrates wax layer and cuticle and then invades the epidermis. The success of this invasion depends on the interaction with the leaf surface (Rumbolz et al*.*, 2000). Therefore, we tested the possibility that the abundance of surface wax might correlate with the susceptibility to Powdery Mildew. We conducted a comparative infection study, determined disease severity for a set of 83 genotypes (Suppl. Fig. S3) and ranked them with respect to their ability to escape symptom development. Low numbers represent a high degree of resistance, high numbers a high degree of susceptibility. We plotted then the ranks obtained for symptom suppression over those obtained for wax abundance (Fig. 5A). For the lower ranks (higher wax levels) up to rank 70, susceptibility was unchanged and relatively low. However, for the higher ranks (lower wax levels), the increase in the rank for wax abundance was accompanied by a concomitant increase of susceptibility. This means that for very thin wax layers Powdery Mildew susceptibility was high, but, as soon as wax abundance exceeded a certain threshold, susceptibility dropped to a residual level that was not lowered further for even thicker wax layers.

We tested further whether the susceptibility for Powdery Mildew differed between the different clades of *V. sylvestris*. However, with exception of clade E, which was significantly more susceptible, we could not find any significant relationship between wax abundance and susceptibility (Suppl. Fig. S4). Thus, the average susceptibility was the same over all clades (Fig. 5B). Also, clade B, which in average had more wax (Fig. 4B) than the other clades, was not more resistant because of this. This was consistent with the observation that susceptibility went up only when the wax layer was below a certain threshold (Fig. 5A).

### Abundant surface wax can interfere with appressorium formation

**Fig. 6:**
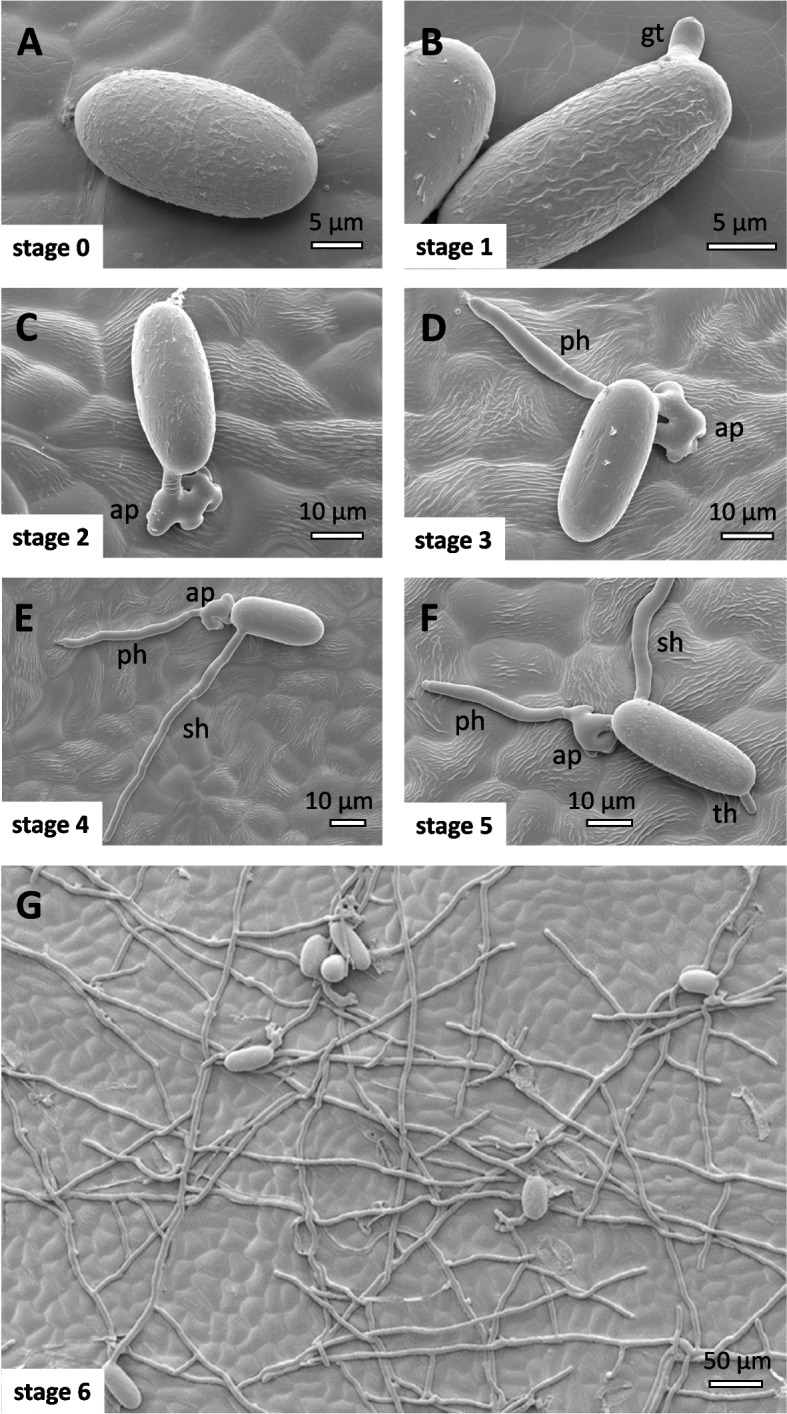
Stageing of Powdery Mildew development on fully expanded leaves of the *vinifera* variety Müller-Thurgau by Cryo-SEM. **A** Stage 0 (ungerminated spores). **B** Stage 1 characterised by the emergence of a germ tube (gt). **C** Stage 2 characterised by the formation of an appressorium (ap). **D** Stage 3 characterised by the outgrowth of a primary hyphae (ph) from the spore at sites different from the appressorium. **E** Stage 4 characterised by the outgrowth of a secondary hyphae (sh) from the appressorium. **F** State 5 characterised by the emergence of tertiary hyphae (th) elsewhere at the spore. **G** state 6 characterised by the formation of a hyphal net on the surface of the leave. Note that in the time scale of the experiment the development only proceeded till stage 3

To understand, why genotypes with abundant surface wax layers were less susceptible to Powdery Mildew, we compared the progress of fungal development over time in ‘Müller-Thurgau’ as commercial variety with a low level of surface wax, and the two *sylvestris* genotypes Ke35 and Ke114 that exhibited abundant surface wax. To quantify the development of *E. necator*, we used the stageing system of Rumbolz et al*.* (2000). The cellular details of these stages were analysed using Cryo-SEM on fully expanded leaves of the variety ‘Müller-Thurgau’ (Fig. 6). The ungerminated spores defined as stage 0, where ovoid and laid loosely on the leaf surface (Fig. 6A). Since they detached easily during processing and fluorescent staining, we did not include them into the quantification. The first developmental event, defined as stage 1, was the emergence of a germ tube at one side of the spores (Fig. 6B). Shortly later, the germ tube became thicker at the distal end and formed the appressorium, which was characteristic of stage 2 (Fig. 6C). After formation of this infection structure, primary hyphae emerged from the spores, usually at sites different from the appressorium, defined as stage 3 (Fig. 6D). The appearance of secondary hyphae from the appressorium was a hallmark for stage 4 (Fig. 6E). Later, tertiary hyphae emerged elsewhere at the spore, defining stage 5 (Fig. 6F). Eventually, a hyphal net developed on the surface of the leaf representing stage 6 (Fig. 6G).

**Fig. 7:**
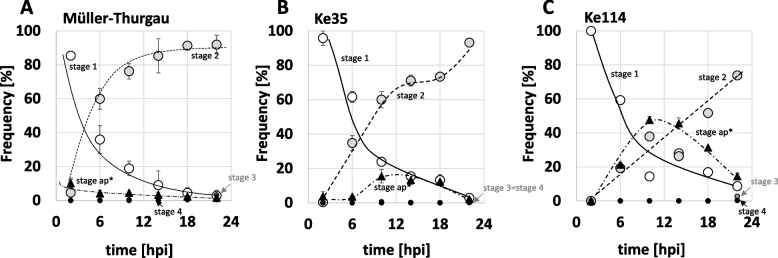
Development of Powdery Mildew on leaves of the *vinifera* variety Müller-Thurgau (**A**) as compared to the *sylvestris* genotypes Ke35 (**B**), and Ke114 (**C**). Frequencies of the different stages as defined in Fig. 6 have been plotted over time in hours post infection (hpi). Data represent mean and standard errors from three biological replicates conducted in fully expanded leaves (#6). Stage ap* represents the aberrant stage found on *sylvestris* leaves, where appressorium development is affected

For a quantitative assessment, we conducted a time-course study labelling the different stages with Fluorescent Brightener 28 comparing the commercial variety ‘Müller-Thurgau’ as low-wax control, comparing to the *sylvestris* genotypes Ke114, accumulating abundant surface wax from early stages of leaf development (Fig. 2F-I) and Ke35, where surface wax was intermediate and developed later (Fig. 2A-D). While the spores of *E. necator* proceeded through the developmental stages, we observed a substantial proportion of spores, where the development of the appressorium was perturbed, such that it did not give rise to hyphae (Suppl. Fig. S4, ap*). When we scored the different stages over time on the three different hosts, we observed distinct differences in progression and amplitude of individual stages (Fig. 7). The development of spores on ‘Müller Thurgau’ (Fig. 7A) proceeded more swiftly than in the two *sylvestris* accessions, as evident from a faster increase in the incidence of stage 2 with complete appressorium formation (Fig. 7B and C). In addition, stage ap* with perturbed appressorium development (Suppl. Fig. S5), which was not seen on ‘Müller-Thurgau’, increased in both *sylvestris* genotypes, albeit transiently. The incidence of this aberrant stage was substantial in Ke114 (where wax abundance was maximal), making up for almost half of the population around 12 hpi. Its prevalence was lower in Ke35 (where wax abundance was lower). We observed that the incidence of aberrant appressoria was transient and decreased after 12 hpi. At the same time, the incidence of normal stage 2 increased (in case of Ke114 visible as a second step-up). Furthermore, the incidence of stage 3 remained low and negligible compared to that of stage 2. The most straightforward scenario for these observations would be that the aberrant appressoria, after some delay, are able to generate hyphae, eventually. Nevertheless, this time-course study shows that wax abundance reduces the efficiency of infection in a dose-dependent manner.

## Discussion

The development of a water impermeable surface belongs to the key innovations that enable the evolutionary success of land plants. In addition to their importance for water autonomy, surface waxes play an important role for the interaction with pathogens. Making use of a germplasm comprising the entire remaining gene pool for the European Wild Grapevine (*Vitis sylvestris*), the ancestor of domesticated Grapevine (*Vitis vinifera*), we investigated the genetic variability of surface wax formation and correlated this with the respective susceptibility to *Erysiphe necator* (the causative agent of Powdery Mildew of Grapevine). Although the *sylvestris* genotypes show more pronounced wax structures as compared to *vinifera* varieties, they are among themselves quite variable with respect to surface wax abundance (in the range of around sixfold between the extreme genotypes). This variability is distributed over different phylogenetic clades of the population, but one of these phylogenetically defined clades shows significantly higher wax abundance compared to the others. Susceptibility to *E. necator* increases significantly when wax abundance drops below a certain threshold. When we follow the development of *E. necator* on leaves with a high surface wax abundant and compare this to the time course on the commercial vinifera variety Müller-Thurgau, we observe that the formation of an appressorium is perturbed and delayed on the high-wax genotypes. It takes > 12 h longer until all attached spores have succeeded to form an appressorium.

These findings lead to the following questions. What mechanisms might be responsible for the delayed appressorium formation and how could this contribute to the partial resistance in those *sylvestris* genotypes? What is the evolutionary context of this phenomenon, since *V. sylvestris* is expected to be a naïve host for *E. necator*, a pathogen originating from North America? How can we find the underlying genetic factors and what can we do with this knowledge? 

### Appressorium Formation – the Achilles Heel of Powdery Mildews

The comparative time courses of fungal development on Müller-Thurgau (low abundance of surface wax) versus the two *sylvestris* genotypes Ke35 and Ke114 (high abundance of surface wax) revealed appressorium formation as crucial step, which was delayed when wax abundance was high (Fig. 7). Among the two *sylvestris* genotypes, the perturbation was more pronounced in Ke114 as compared to Ke35, correlated with a wax abundance that was maximal in Ke114. This suggests that surface waxes are a factor that can interfere with fungal development. The appearance of aberrant appressoria was a transient phenomenon, though. Eventually, the pathogen was able to re-enter development. However, the delay due to the aberrant appressorium was substantial, in the range of ~ 12 h, which would be a sufficient time frame to employ defence reactions of the host.

One possible mechanism might be that the hydrophobicity of abundant surface waxes might impede fungal development. This has been proposed for the related species *E. graminis*, infecting Poaceaen hosts. For the abaxial side of leaves of *Lolium perenne*, where appressorium formation and penetration structures are aborted, linked with presence of wax plates, fungal development can be restored by removing these epicuticular waxes [45]. These waxes might impede appressorium formation merely by their hydrophobicity, as suggested by comparative approaches of fungal development on leaves as compared to artificial surfaces [46].

Alternatively, they might impede the diffusion of chemical signals from the cuticle that are sensed by the fungus to steer its development. Such plant-derived compounds that act as guiding signals for fungal development are well known from the interaction with Rust fungi (for a classical review see [47]). This alternative hypothesis that the protective function of surface waxes against Powdery Mildews is linked to interception of such signalling is supported by a growing body of evidence, mainly from Powdery Mildews of Gramineae (for a recent review see [48]). For instance, hexacosanal, a long-chained aldehyde (C_26_) was found to strongly stimulate appressorium formation in *E. graminis* [49]. This is supported by findings on the maize *glossy11* mutant completely lacking very-long chained cuticular aldehydes, where fungal development is aborted, but can be rescued by spraying hexacosanal [50]. Along similar lines, suppression of activators for very-long chain aldehyde biosynthesis blocks germination of Wheat Powdery Mildew [51]. The fact that we do find only a partial correlation between wax abundance and resistance to Powdery Mildew, indicates that chemical composition plays a role as well, an aspect that should be addressed in future research.

Our data are congruent with a comparative study conducted on berries from different *vinifera* varieties, wild North American species, and hybrids thereof [52], where the thickness of the wax layer correlated well with post-harvest resistance against *Botrytis cinerea*, the causative agent of Grey Mold. With respect to *E. necator*, a similar study detected a sharp decline in infection once the berry skin developed a wax layer [53]. For the same fungus, the receptor, a so-called signalling mucin, has been identified using respective mutants. This receptor is a transmembrane protein, which protrudes into the apoplast with a large O-glycosylated domain sensing components of the plant cell wall and then initiating appressorium formation through a MAPK cascade [54]. A well-developed wax-layer would interfere the access of host-cell wall factors to the mucin and, thus, halt fungal development. To what extent such a working model can be transferred to *E. necator*, remains to be elucidated. There is evidence, though, supporting such a mechanism for *E. necator*. The above-mentioned age-dependent resistance of older berries [53] goes along with arrest of fungal development before any penetration pore is formed [55], indicating that the wax layer interferes with a signal needed for appressorium formation.

### Is Wax Formation an Exaption of *V. sylvestris*?

Resistance genes from North American wild grapes corresponding to the second, specific layer of plant innate immunity [56, 57] have been central for the breeding of novel grapevine varieties that can cope with *E. necator* [21]. Such specific forms of immunity require a co-evolutionary history between host and pathogen. It was, therefore, a surprise, when resistance loci against Powdery Mildew were discovered in wild grapes from China [14] as well as in Usbek *vinifera* varieties [58]. While these genetic factors are extremely useful for gene stacking of resistance factors, claims that they are due to effector-triggered immunity have to be seen with scepticism (for a detailed discussion see [59]). Similarly, the partial resistance to Powdery Mildew found during the current study in European Wild Grapevines should not be interpreted as adaptive trait against *E. necator*, since these grapevines are naïve hosts, lacking any evolutionary relationship with the pathogen whatsoever. Thus, surface waxes in *V. sylvestris* represent a clear case of exaptation *in *sensu Gould and Vrba [60], meaning that the selective advantage conferred by this trait derives from a functional shift caused by changes in the environment, not by changes in the genetics of the organism.

This poses the question, what the adaptive context for the thicker wax layers might be. Reduced transpiration would rather be associated with intra-cuticular waxes [61]. Moreover, the stomata of *V. sylvestris* are protruding [62] indicating that transpiration control is not a major selective factor in the Wild European Grapevine, consistent with its ecological niche, alluvial forests that are regular flooded. Thus, the function of the pronounced epicuticular waxes is more likely to be sought in constraining microbial colonisation of the leaf surface, similar as for the *Lotus* effect [33], by the way, *Nelumbo nucifera* as well is inhabitating humid ecosystems. In fact, the fungal biodiversity on leaves of *V. vinifera* is considerable and includes numerous saprotrophes, but also necrotrophic pathogens [63]. Thus, the most likely scenario is that the pronounced surface waxes confer a selective advantage in the humid environment of the alluvial forest because they constrain the colonisation by saprotrophic and necrotrophic microbes and support self-cleaning. The effect that this wax layer also prevents diffusion of host-derived factors guiding appressorium formation in *E. necator* represents a by-product of these surface waxes but was not a selective factor driving their evolution. Thus, with respect to *E. necator*, the surface wax layers in *V. sylvestris* must be considered as pre-adaptive trait. It will be interesting in the future to investigate the fungal microbiome on these leaves as compared to *vinifera* varieties with their only residual wax layers. To what extent wax composition is of relevance here, poses a further rewarding question for future research – since the fungi use specific chemical cues from the host surface as signals to steer their development, the possibility that the surface waxes in *V. sylvestris* lack these signals cannot be ruled out.

### How to find wax genes and what to do you with them

Wax synthesis and deposition are expected to depend on many factors including the activity of metabolic enzymes such as elongases [37], or the terminal decarboxylase CYP96A15 [36], but also the abundance of transporters, the intensity of vesicle trafficking or the availability of Lipid Transfer Proteins. While it cannot be excluded that the high abundance of surface waxes found in clade B of the *sylvestris* population (Fig. 4B) might originate from a genetic difference that is synapomorphic for this group, it is more likely that different genotypes accumulate surface waxes due to different genetic factors. Basically, two strategies can be conceived – a non-biased Genome Wide Association Study (GWAS) to detect genetic associations on the one hand, or a targeted search for candidate genes. The genome database established for the *sylvestris* collection addressed in this study [44] can facilitate both approaches. Currently, we are addressing function and regulation of the grapevine homologues for two MYB transcription factors that regulate metabolic enzymes in VLFA synthesis [64]. Validated genetic factors contributing to surface wax abundance can then be introgressed into *vinifera* varieties of interest using a marker-assisted selection strategy. Since these factors contribute to resistance against Powdery Mildew by a mechanism that differs from the ETI-based immunity from North American wild grapevines, they can be useful for gene stacking [21] leading to a sustainable resistance that cannot easily be breached by pathogen strains that overcome R-gene mediated resistance.

## Conclusions

In the current study, we addressed the question, whether the partial resistance against the grapevine pathogen *E. necator*, the causative agent for Powdery Mildew, found in *V. sylvestris*, the ancestor of domesticated Grapevine, *V. vinifera*, might be linked with the more pronounced formation of surface waxes on the leaves. We compared the abundance of surface waxes across a germplasm collection comprising the entire known gene pool for this wild ancestor having survived in Germany using CryoSEM in combination with quantitative image analysis and project these data on the molecular phylogeny for *V. sylvestris* based on whole-genome sequencing [44]. While we find considerable genetic variation between different clades of *V. sylvestris*, we observe that most wild accessions accumulate more wax than ‘Müller-Thurgau’ a commercial *vinifera* variety, common in South Germany. Among the *sylvestris* accessions, clade B accumulates significantly more wax than the other clades. When we screened the germplasm for susceptibility against *E. necator*, using controlled inoculation, we see an elevated susceptibility, when surface-wax abundance drops below a certain threshold. This non-linear relationship indicates that in addition to abundance, other factors, such as chemical composition, might be relevant, an aspect that should be addressed in future studies. When we follow early infection on hosts differing in wax deposition, we find that the formation of an appressorium becomes perturbed and delayed on hosts with strong wax deposition, which is not seen on ‘Müller-Thurgau’, which is depleted in surface waxes. Thus, surface waxes can improve the performance under infection pressure, such that introgression of the underlying genetic factors into commercial grapevine varieties would be a feasible strategy to improve resistance to Powdery Mildew while reducing the need to apply fungicides.

## Materials and methods

### Plant material

This study used the germplasm collection established in the Botanical Garden of the Karlsruhe Institute of Technology and comprised 102 genotypes of *Vitis vinifera ssp. sylvestris*. These included the last viable German population for this species at Ketsch peninsula in an alluvial forest at the banks of the Rhine River between Karlsruhe and Mannheim along with a few residual individuals from other sites of the Upper Rhine valley. In addition, 8 V*. vinifera ssp. vinifera* varieties were included into the study that are either common in German and French vineyards, along with 5 traditional landraces from Central Europe, Tunisia, and Teneriffa (Canary Archipelago). The majority of these genotypes have been fully sequenced [44], and their phylogenetic relationships assesses based on maximum-likelihood clustering based on genome-wide single-nucleotide polymorphisms, which allows to group the *sylvestris* population into five clades (Suppl. Fig. S2). Identity, accession codes, and origin of the individual accessions used in this study are compiled in Suppl. Table 1.

### Cryo-Scanning electron microscopy analysis (cryo-SEM)

Small leaf samples of about 5 mm × 5 mm were excised from freshly harvested leaves and immediately fixed on a plane cryo transfer shuttle with conductive mounting medium (1:1 mix of Tissue-Tek and colloidal graphite, Agar Scientific Ltd., Stansted, United Kingdom) and then processed according to [65]. In brief, after shock-freezing at -210 °C in nitrogen slush, the samples were transferred to a pre-cooled (-135 °C) cryo chamber (PP2000 T, Quorum Technologies Ltd., Laughton, United Kingdom) and sublimated for 15 min at -90 °C for 15 min. After sputtering with platinum (30 s coating at 5–10 mA in an Argon atmosphere), the specimens were transferred to the cryo-stage in the SEM chamber (T = -135 °C) and imaged (Quanta 250 FEG field emission scanning electron microscope, FEI, Brno, Czech Republic) under ultravacuum (3 10^–7^ mbar). Backscattered electrons were collected by an Everhart–Thornley detector at a working distance of 5 mm, and an accelerating voltage of 10 kV.

### Quantification of wax structures

We measured the wax structures on the upper surface of grapevine leaves by quantitative image analysis from the digital images obtained by scanning electron microscopy using the freeware ImageJ (https://imagej.nih.gov/ij/). After conversion into binary images, we inverted them, such that the wax structures appeared black on a white background (Suppl. Fig. S1B). The Analyze Particle tool enabled then automatic detection based on their size and circularity. To exclude background noise, the minimal level for detection was set to 1 square pixels, to exclude unspecific particles, such as dust on the leaf surface, the maximal level for detection was set to 1000 square pixels. A circularity filter of 0–0.2 was then used to select elongated structures (waxwings and ribs), a circularity filter of 0.2–1 selected rounder structures, such as wax crystals. For each circularity setting, we recorded the total area of the selected structures and calculated the ratio with the total area of the ROI to estimate the wax coverage. Each data point represents three independent leaves. It should be noted that, due to the binarisation, this quantification strategy does not yield absolute values. It can be used to compare wax abundance within an experimental series but cannot be used to draw comparisons across different series conducted, for instance, in different years.

### Inoculation with Erysiphe necator

For infection with *Erysiphe necator* (the causative agent of Powdery Mildew of Grapevine) a field isolate was used that had been sampled from an affected vineyard in Neustadt an der Weinstraße (Palatinate, Germany). This isolate had been maintained on the susceptible variety *V. vinifera ssp. vinifera* cv ‘Müller-Thurgau’ in the greenhouse of the DLR Rheinland-Pfalz (Neustadt an der Weinstraße) by regularly placing recipient plants between heavily infected donor plants. For controlled inoculation, fresh, fully expanded leaves from infested plants were collected at sporulation as source for *E. necator* conidia. To follow the time course of colonisation, leaf discs were excised from fully expanded leaves of either the susceptible *V. vinifera ssp. vinifera* cv. ‘Müller-Thurgau’, and two representative genotypes of *V. vinifera ssp. sylvestris*, Ke35 (belonging to clade C of the Ketsch population) and Ke114 (belonging to clade D of the Ketsch population), and maintained on wet filter paper in Petri dishes (94 mm diameter, 16 mm height; Greiner, Kremsmünster, Austria to ensure full turgescence. To achieve a homogenous distribution, a paper cylinder of 60 cm height was placed on top of the leaf discs, and leaves of the infested donor plant was shaken above the cylinder. To prevent the inoculated leaf discs from desiccation, the Petri dish was closed and wrapped by Nescofilm. Then the specimen was incubated in a climate chamber (Percival Scientific, CFL PlantClimatics, Wertingen; Germany) with a temperature of 24 °C under a 16 h light/8 h dark cycle (120 μmol^.^m^–2^ s^−1^ white light. The leaf discs were sampled at 2, 6, 10, 14, 18, and 22 h post inoculation (hpi). Data represent mean and standard error from three independent experimental series with three leaf discs from three individuals.

### Microscopic analysis and stageing of *E. necator* development

The leaf discs were transferred directly, abaxial side down, on a slide with around 2–3 drops of 0.1% w/v Fluorescent Brightener 28 (Sigma-Aldrich, Deisenhofen, Germany) in 50 mM Tris HCl (pH 9) complemented with 01% v/v Tween 20 and incubated 10–20 min at room temperature. After rinsing off excess dye with buffer, the specimen was ready for analysis by fluorescence microscopy (Apotome, Zeiss, Jena) after adding sufficient buffer from side of the cover slip to allow the leaf disc to assume a flat shape. The conidia on the leaf disc were examined using excitation at 450–490 nm, a beamsplitter at 515 nm, and a long-pass emission filter > 520 nm). For the classification of conidia we used a stageing system [66, 67] as illustrated in Suppl. Fig. S4 and Fig. 6. Stage 0 comprised ungerminated spores. As stage 1, we defined spores with initiated germ tubes. Stage 2 were spores, which had developed an appressorium. Stage 3 was covering spores that had generated a first hypha. Stage 4 were spores, where a secondary hypha had appeared. Stage 5 corresponded to spores, where a tertiary hypha had emerged. Stage 6 stood for full mycelial coverage. On the sylvestris hosts, we observed an additional stage ap* with spores with aberrant or absent appressorium.

### Estimation of susceptibility to *E. necator*

For each genotype, at least three plants were scored for their susceptibility to *E. necator* using spontaneous infection by placing the test plants between heavily infested grapevines in the greenhouse. After three weeks, severity of infestation was evaluated based on the coverage of the infected leaf areas using the rating scheme as described in the EPPO guideline PP 1/4 (4) for *Erysiphe necator* (Suppl. Table 2). The median of infestation values recorded for the ten youngest fully developed leaves was used as readout for the respective individual. For each experiment, three individuals of the highly susceptible *vinifera* variety 'Müller-Thurgau' served as a positive control.

### Data analysis

Since the quantification of wax abundance by quantitative image analysis yields only relative values, the results can only be compared within a given experimental series, and not transferred between different measuring campaigns, conducted, for instance, in different years. Moreover, the values for wax abundance are skewed and do not follow a normal distribution. The same holds true for the data on susceptibility to Powdery Mildew. We used, therefore, statistical methods based on the rank of a data point, rather than its absolute value. Consequently, significance of difference was tested using the non-parametrical Kruskal–Wallis test in Figs. 4 and 5, and correlations between wax abundance and susceptibility were inferred using the non-parametrical Spearman correlation test.

## Supplementary Information

**Additional file 1: Figure S1.** Quantification of surface wax from SEM images. **Figure S2.** Phylogenetic relationship of the sylvestris accessions. **Figure S3.** Disease severity over the sylvestris population. **Figure S4.** Relationship between susceptibility to Powdery Mildew and abundance of surface waxes in the sylvestris population. **Figure S5.** Stageing of Powdery Mildew development. **Table S1.** Identity and origin of the grapevine accessions. **Table S2.** Evaluation of infestation with Powdery Mildew.

## Data Availability

The datasets used and analysed during the current study available from the corresponding author on reasonable request.

## Abbreviations

VLCFAs: Very Long Fatty Acids
LTPs: Lipid Transfer Proteins
CryoSEM: Cryo Scanning Electron Microscopy
