# Supplementary material for: Surface wax in the ancestral grapevine Vitis sylvestris correlate with partial resistance to Powdery Mildew
Source: BMC Plant Biol. 2023 Jun 7;23:304. doi: 10.1186/s12870-023-04311-x (PMC10245546; doi:10.1186/s12870-023-04311-x)
Supplement: Supplementary file 1 — Additional file 1: Figure S1. Quantification of surface wax from SEM images. Figure S2. Phylogenetic relationship of the sylvestris accessions. Figure S3. Disease severity over the sylvestris population. Figure S4. Relationship between susceptibility to Powdery Mildew and abundance of surface waxes in the sylvestris population. Figure S5. Stageing of Powdery Mildew development. Table S1. Identity and origin of the grapevine accessions. Table S2. Evaluation of infestation with Powdery Mildew. [file 12870_2023_4311_MOESM1_ESM.pdf]

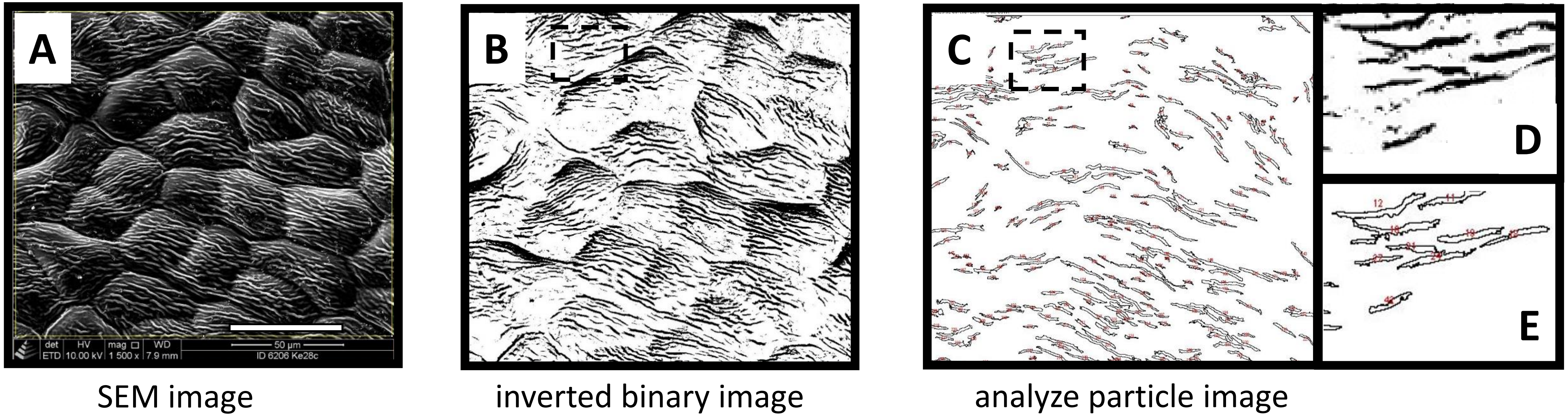

Supplementary Figure S1. Quantification of surface wax from SEM images. **A** Original SEM image of a grapevine leaf surface with wax structures. **B** Inverted binary image used for automatic detection. Inset refers to the zoom-in in **D**, **C** Automatic detection of surface wax structures using the Analyse Particle tool, the inset refers to the zoom-in in **E**. **D** and **E** show a zoom in of the inverted binary image and the automatically recognised wax structures used for quantification.

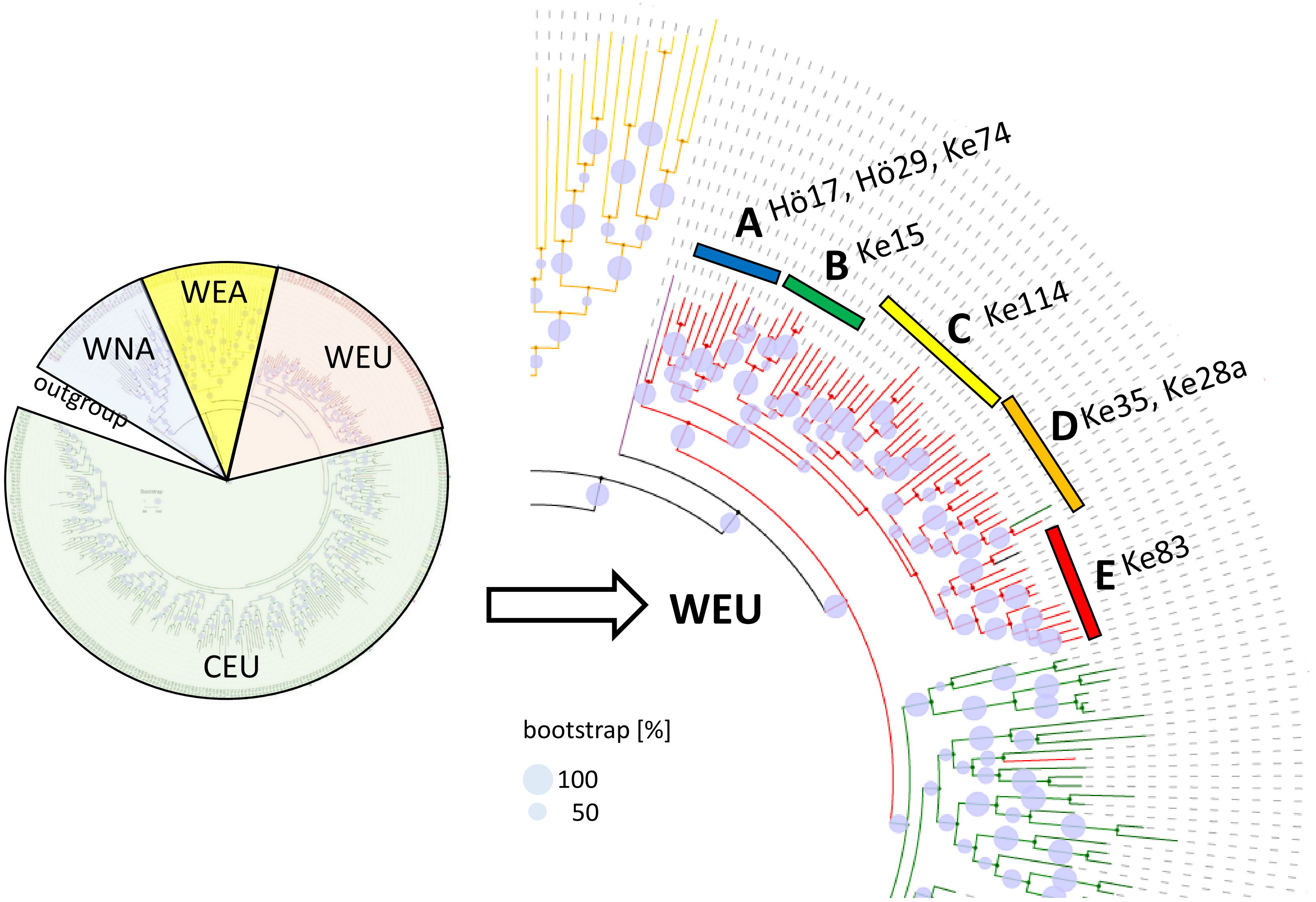

Supplementary Figure S2. Phylogenetic relationship of the *sylvestris* accessions inferred from whole-genome sequencing by Maximum Likelihood (from Liang et al. 2019) and position of the five clades and the representative genotypes shown in **Figure 3**.

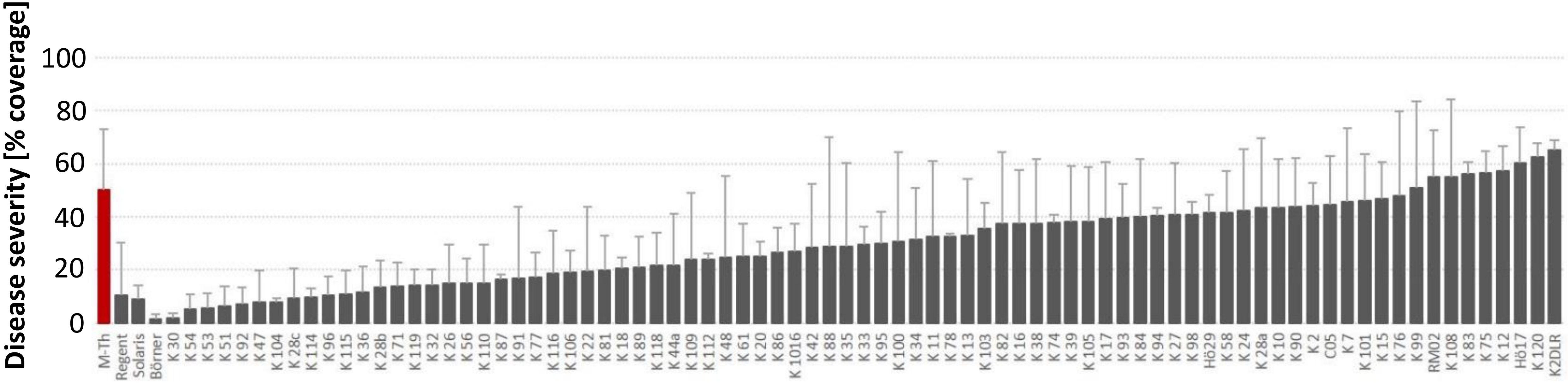

Supplementary Figure S3. Disease severity after standardised infection with Erysiphe necator as scored by the EPPO PP Directive 1/4 based on leaf coverage with sporangia [%]. Data represent mean and standard error from three independent experimental series with three leaf discs from three individuals.

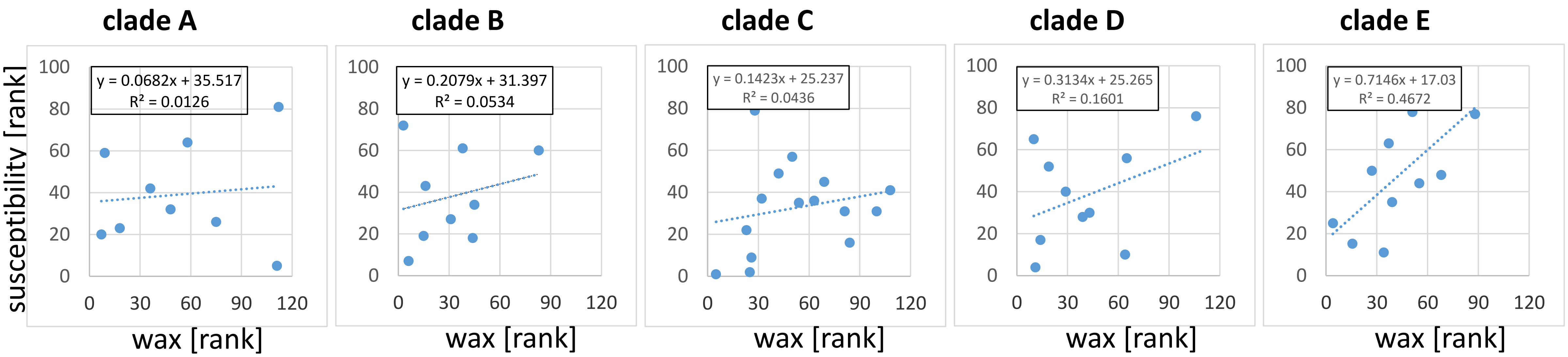

Supplementary Figure S4. Relationship between susceptibility to Powdery Mildew and abundance of surface waxes in the *sylvestris* population broken down for the different clades individually using a ranking system. For each genotype, the rank in susceptibility (increasing numbers mean increasing susceptibility) and wax abundance (increasing numbers mean decreasing wax abundance) were determined and plotted.

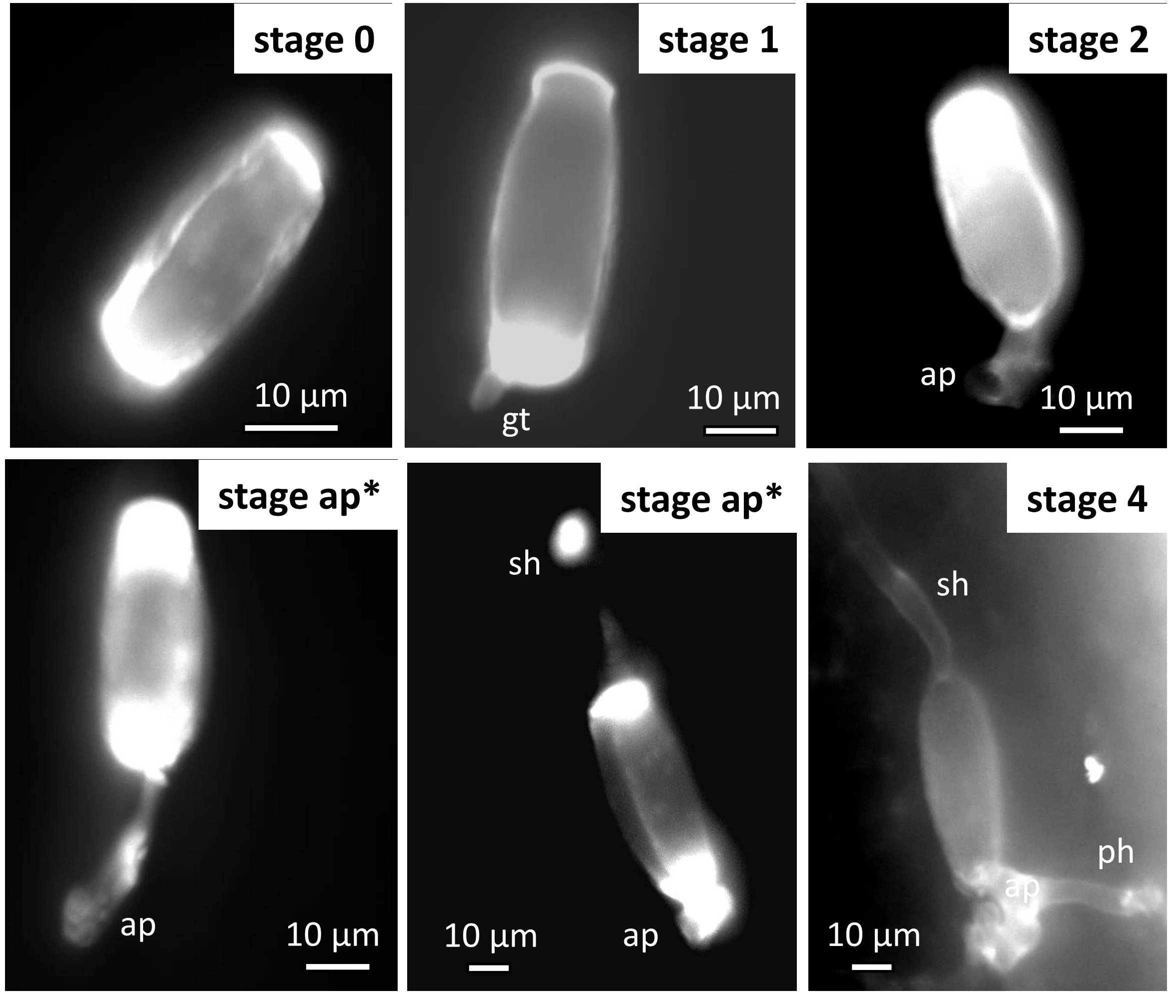

Supplementary Figure S5. Stageing of Powdery Mildew development on fully expanded leaves of *V. sylvestris* assessed by fluorescence microscopy after staining with Fluorescent Brightener 28. Stage 0 (ungerminated spores), stage 1 characterised by the emergence of a germ tube (gt), and stage 2 characterised by the formation of an appressorium (ap) could be observed, while stage 3 characterised by the outgrowth of a primary hyphae from the spore at sites different from the appressorium was not observed, instead, an aberrant stage ap* was found, where appressorium is perturbed (left image), stage 4 with both hyphae being present, could be observed.

Supplementary Table 1. Identity and origin of the grapevine accessions used in this study

Taxon	Voucher ID	Source	Origin
Vitis vinifera ssp. vinifera			
**Commercial cultivars**			
cv. Müller-Thurgau	5585	WBI Freiburg	Economically important German variety
cv. Cabernet Sauvignon	7471	JKI Siebeldingen	Economically important French variety
cv. Pinot Noir	7474	JKI Siebeldingen	Economically important traditional French variety, background of genome project
cv. Riesling	6144	WBI Freiburg	Traditional German variety
cv. Gewürztraminer	7464	Antes Nursery	Traditional Alsacian variety
cv. Chardonnay	7472	JKI Siebeldingen	Traditional French variety
**Hybrid varieties**			
cv. Merzling	6150	WBI Freiburg	German variety, pedigrée of North American and Chinese species
cv. Regent	5895	JKI Siebeldingen	German variety, pedigrée of North American species
**Traditional landraces**			
cv. Razegui	5894	Borj Cedria	Tunisian landrace
cv. Augster Weiß	7443	JKI Siebeldingen	Central European medieval landrace, male sterile
cv. El Tanque	5898	Tenerife, Canarian Islands	Canarian landrace
cv. Guimar	5899	Tenerife, Canarian Islands	Canarian landrace
cv. San Andres	5889	Tenerife, Canarian Islands	Canarian landrace
Vitis vinifera ssp. sylvestris			
**Isolated, Upper Rhine**			
VSylE	5905	WBI Freiburg	Alsace, near Colmar
Co5	6186	DLR Neustadt	Alsace, near Colmar
Klaus	6173	Dr. Schubert	Reiß Island, near Mannheim
Blau	6183	Dr. Schubert	Reiß Island, near Mannheim
Rosa	6185	Dr. Schubert	Reiß Island, near Mannheim

Hö17	6187	Own collection	Hördt, Rhine bank
Hö29	6188	Own collection	Hördt, Rhine bank
**Ketsch population, Rhine**			
Ke 2	6189	Own collection	Ketsch peninsula, Rhine bank
Ke 7	6190	Own collection	Ketsch peninsula, Rhine bank
Ke 10	6191	Own collection	Ketsch peninsula, Rhine bank
Ke 11	6192	Own collection	Ketsch peninsula, Rhine bank
Ke 12	6193	Own collection	Ketsch peninsula, Rhine bank
Ke 13	6194	Own collection	Ketsch peninsula, Rhine bank
Ke 15	6195	Own collection	Ketsch peninsula, Rhine bank
Ke 16	6196	Own collection	Ketsch peninsula, Rhine bank
Ke 17	6197	Own collection	Ketsch peninsula, Rhine bank
Ke 18	6198	Own collection	Ketsch peninsula, Rhine bank
Ke 20	6199	Own collection	Ketsch peninsula, Rhine bank
Ke 22	6200	Own collection	Ketsch peninsula, Rhine bank
Ke 24	6201	Own collection	Ketsch peninsula, Rhine bank
Ke 26	6202	Own collection	Ketsch peninsula, Rhine bank
Ke 27	6203	Own collection	Ketsch peninsula, Rhine bank
Ke 27 neu	8618	Own collection	Ketsch peninsula, Rhine bank
Ke 28a	6204	Own collection	Ketsch peninsula, Rhine bank
Ke 28b	6205	Own collection	Ketsch peninsula, Rhine bank
Ke 28c	6206	Own collection	Ketsch peninsula, Rhine bank
Ke 30	6207	Own collection	Ketsch peninsula, Rhine bank
Ke 32	6208	Own collection	Ketsch peninsula, Rhine bank
Ke 33	6209	Own collection	Ketsch peninsula, Rhine bank
Ke 34	6210	Own collection	Ketsch peninsula, Rhine bank
Ke 35	6211	Own collection	Ketsch peninsula, Rhine bank
Ke 36	6212	Own collection	Ketsch peninsula, Rhine bank
Ke 38	6213	Own collection	Ketsch peninsula, Rhine bank
Ke 39	6214	Own collection	Ketsch peninsula, Rhine bank
Ke 42	6215	Own collection	Ketsch peninsula, Rhine bank
Ke 44a	6216	Own collection	Ketsch peninsula, Rhine bank
Ke 47	6218	Own collection	Ketsch peninsula, Rhine bank
Ke 48	6219	Own collection	Ketsch peninsula, Rhine bank
Ke 51	6220	Own collection	Ketsch peninsula, Rhine bank
Ke 53	6221	Own collection	Ketsch peninsula, Rhine bank
Ke 54	6222	Own collection	Ketsch peninsula, Rhine bank
Ke 56	6223	Own collection	Ketsch peninsula, Rhine bank
Ke 58	6224	Own collection	Ketsch peninsula, Rhine bank
Ke 61	6225	Own collection	Ketsch peninsula, Rhine bank
Ke 71	6226	Own collection	Ketsch peninsula, Rhine bank
Ke 74	6227	Own collection	Ketsch peninsula, Rhine bank

Ke 75	6228	Own collection	Ketsch peninsula, Rhine bank
Ke 76	6229	Own collection	Ketsch peninsula, Rhine bank
Ke 77	6230	Own collection	Ketsch peninsula, Rhine bank
Ke 78	6231	Own collection	Ketsch peninsula, Rhine bank
Ke 81	6233	Own collection	Ketsch peninsula, Rhine bank
Ke 82	6234	Own collection	Ketsch peninsula, Rhine bank
Ke 83	6235	Own collection	Ketsch peninsula, Rhine bank
Ke 84	6236	Own collection	Ketsch peninsula, Rhine bank
Ke 86	6237	Own collection	Ketsch peninsula, Rhine bank
Ke 87	6238	Own collection	Ketsch peninsula, Rhine bank
Ke 88	6239	Own collection	Ketsch peninsula, Rhine bank
Ke 89	6240	Own collection	Ketsch peninsula, Rhine bank
Ke 90	6241	Own collection	Ketsch peninsula, Rhine bank
Ke 91	6242	Own collection	Ketsch peninsula, Rhine bank
Ke 92	6243	Own collection	Ketsch peninsula, Rhine bank
Ke 93	6244	Own collection	Ketsch peninsula, Rhine bank
Ke 94	6245	Own collection	Ketsch peninsula, Rhine bank
Ke 95	6246	Own collection	Ketsch peninsula, Rhine bank
Ke 96	6247	Own collection	Ketsch peninsula, Rhine bank
Ke 98	6248	Own collection	Ketsch peninsula, Rhine bank
Ke 99	6249	Own collection	Ketsch peninsula, Rhine bank
Ke 100	6250	Own collection	Ketsch peninsula, Rhine bank
Ke 101	6251	Own collection	Ketsch peninsula, Rhine bank
Ke 1016	6252	Own collection	Ketsch peninsula, Rhine bank
Ke 103	6253	Own collection	Ketsch peninsula, Rhine bank
Ke 104	6254	Own collection	Ketsch peninsula, Rhine bank
Ke 106	6256	Own collection	Ketsch peninsula, Rhine bank
Ke 107	6257	Own collection	Ketsch peninsula, Rhine bank
Ke 108	6258	Own collection	Ketsch peninsula, Rhine bank
Ke 109	6259	Own collection	Ketsch peninsula, Rhine bank
Ke 110	6260	Own collection	Ketsch peninsula, Rhine bank
Ke 112	6261	Own collection	Ketsch peninsula, Rhine bank
Ke 114	6262	Own collection	Ketsch peninsula, Rhine bank
Ke 115	6263	Own collection	Ketsch peninsula, Rhine bank
Ke 116	6264	Own collection	Ketsch peninsula, Rhine bank
Ke 118	6265	Own collection	Ketsch peninsula, Rhine bank
Ke 119	6266	Own collection	Ketsch peninsula, Rhine bank
Ke 120	6267	Own collection	Ketsch peninsula, Rhine bank
K2 DLR	6268	DLR Neustadt	Ketsch peninsula, Rhine bank
RM 2	6269	DLR Neustadt	Ketsch peninsula, Rhine bank
VSylJung-1	7778	Dr. Jung	Ketsch peninsula, Rhine bank
VSylJung-2	7779	Dr. Jung	Ketsch peninsula, Rhine bank
Ke 23-ITA	8323	JKI Siebeldingen	Ketsch peninsula, Rhine bank
KE 006-GSH	8324	Geisenheim	Ketsch peninsula, Rhine bank

VvsBgFfm	8581	BG Frankfurt	Ketsch peninsula, Rhine bank
**Isolated, Danube**			
Ö3	6174	Naturgarten	Lobau, Danube bank, Austria
Ö5	6175	Naturgarten	Lobau, Danube bank, Austria
Ö6	6176	Naturgarten	Lobau, Danube bank, Austria
Ö7	6177	Naturgarten	Lobau, Danube bank, Austria
Ö8	6178	Naturgarten	Lobau, Danube bank, Austria
Ö9	6179	Naturgarten	Lobau, Danube bank, Austria
Ö10	6180	Naturgarten	Lobau, Danube bank, Austria
Ö11	6181	Naturgarten	Lobau, Danube bank, Austria
Ö12	6182	Naturgarten	Lobau, Danube bank, Austria
VsylRO-01	6556	Prof. Dr. Dister	Romania, Danube bank

Supplementary table 2. Evaluating the degree of infestation of Powdery Mildew in plant according to the EPPO PP Directive 1/4 (4) *Erysiphe necator*, modified.

category [%]	0	2.5	7.5	17.5	37.5	62.5	82.5	95
description [%]	0	>0-5	5-10	10-25	25-50	50-75	75-90	90-100
